# Photoprotective and Antioxidant Liposomal Formulations from *Sargassum*-Derived Biomolecules for Skincare Applications

**DOI:** 10.1021/acsomega.5c09406

**Published:** 2025-10-25

**Authors:** Angela Cruz-Lugo, Sofía V. Rivera-Santiago, Ana S. Cortés-Norat, Valeria I. Rosario-Isona, Sandra V. Nieves-Moron, Victoria V. Viera-Sánchez, Liz M. Díaz-Vázquez

**Affiliations:** 19878 University of Puerto Rico, Río Piedras Campus, San Juan 00925-2537, Puerto Rico

## Abstract

The development of sustainable skincare formulations is increasingly important due to growing customer demand for eco-friendly and health-conscious products. Conventional formulations often rely on synthetic ingredients that can harm human health and marine ecosystems, underscoring the need for natural and biodegradable alternatives. Recurrent *Sargassum* blooms in tropical regions generate vast, underutilized biomass, posing environmental and economic challenges, such as habitat disruption and waste accumulation. Valorizing this biomass for nanocarrier production aligns with circular economic principles and promotes marine resource utilization. This study developed and characterized fucoidan-coated liposomes as nanocarriers for skincare applications through comprehensive physicochemical and functional evaluations. Lipids extracted from *Sargassum* were combined with fucoidan, a sulfated polysaccharide, using the thin-film hydration technique in a 1:1 v/v ratio. Treatments included empty and fucoidan-coated liposomes, assessed for particle size, zeta potential, thermal stability, photodegradation resilience, and antioxidant activity. Fucoidan-coated liposomes exhibited a particle size of 673 ± 10 nm, a PDI of 0.8, and a zeta potential of −72 mV, maintaining colloidal stability for 28 days. FTIR confirmed structural integrity under simulated sunlight, while TGA, DSC, and gas chromatography showed improved lipid retention and thermal stability. Although empty liposomes exhibited stronger initial radical scavenging activity (0.9 ± 0.4 g/L), fucoidan-coated liposomes (2.0 ± 0.6 g/L) provided more sustained antioxidant effects due to the protective polysaccharide layer. This approach integrates marine biomass valorization with enhanced liposomal stability and antioxidant performance, positioning fucoidan-coated *Sargassum* liposomes as a robust and eco-friendly platform for next-generation skincare formulations within a circular economy framework.

## Graphical abstract

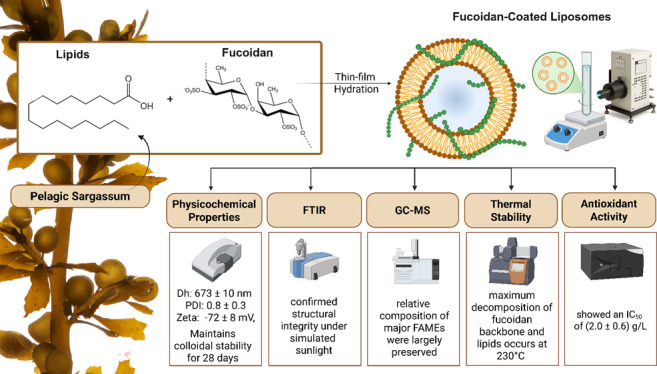

## 1 Introduction

As humanity confronts the dual frontiers of climate change and space exploration, the demand for innovative skincare that can protect and adapt to extreme environments has never been greater. Rising ultraviolet (UV) radiation from ozone depletion threatens not only ecosystems but also human skin, the body’s first line of defense, and disrupts cellular growth, metabolism, and repair.^1^ Astronauts, in turn, encounter microgravity, radiation, and psychological stressors that intensify dermatological problems.^2,3^ Sunscreen use has also become imperative for protecting skin from the harmful effects of UV radiation. Sunscreens, formulated from chemical agents such as avobenzone and oxybenzone or physical agents such as zinc oxide and titanium dioxide, remain the primary defense. Despite their widespread use, concerns have emerged regarding human health and environmental safety. Oxybenzone and avobenzone are associated with endocrine disruption, photoallergic reactions, and systemic absorption in humans. In contrast, zinc oxide and titanium dioxide nanoparticles may generate reactive oxygen species or penetrate compromised skin.^4,5^ From an ecological perspective, sunscreen residues accumulate in aquatic environments, where they have been linked to coral bleaching, bioaccumulation, and long-term toxicity in marine organisms.^4,6,7^

To address the damaging effects associated with physical and chemical sunscreens, there is an urgent need for safer and more natural options. Recent studies on plant-derived polysaccharides, such as those extracted from *Christia verpertilionis* and *Alocasia cucullata*, have revealed significant bioactivities, including antitumor and immunomodulatory effects, as well as favorable physicochemical properties that support their use in skincare applications.^8,9^ Macroalgae have garnered increased global attention as promising sources of bioactive compounds for skincare applications due to their resilience to extreme environmental conditions. These vital marine sources are recognized for their abundance of polysaccharides, polyphenols, and others with antioxidant and photoprotective properties.^10,11^ Studies have demonstrated that sulfated polysaccharides, such as fucoidan derived from brown algae, possess potent radical scavenging and anti-inflammatory properties, making them attractive candidates for skincare formulations.^12^ Similarly, algal lipids and carotenoids are being investigated for their potential to improve skin hydration and protect against photoaging.^13,14^

In 2025, nearly 38 million metric tons of *Sargassum* were detected across the Caribbean and adjacent Atlantic zones, far more than in past years.^15^ Stench, beach closures, cleanup costs, and ecosystem degradation disrupt tourism, aquaculture, wildfire, and human health.^16,17^ While many studies emphasize the valorization of *Sargassum* primarily to reduce coastal pollution and ecological harm, fewer investigate its skin-beneficial bioactives, such as antioxidants and anti-inflammatory compounds, and their mechanistic potential for dermal health.^18^ In parallel, consumer demand for eco-friendly and clean beauty is expanding rapidly, with over 70% of consumers favoring brands that demonstrate sustainable practices, alongside a growing global trend toward the use of natural and organic products in the beauty industry.^19–21^ Moreover, the market for marine-derived ingredients is confirmed by the increasing production and growing willingness of consumers to buy these products.^22^ Together, these dynamics suggest that linking the biochemical efficacy of *Sargassum* with clear evidence of market demand and consumer preference for eco-friendly skincare strengthens the need for its adoption in the skincare industry.

Fucoidan, a sulfated polysaccharide found in *Sargassum*, possesses anti-inflammatory, antioxidant, UV-protective, and anticoagulant effects.^23^ Fucoidan can also improve skin health and help prevent or alleviate signs of aging, such as dullness, dryness, dark spots, and wrinkles.^24^ Adding fucoidan to skin care formulations can enhance dermal fibroblast proliferation and collagen deposition, with no discernible odor or taste.^25^ Compared to other marine-derived polysaccharides, such as chitosan and alginate, fucoidan offers distinct advantages: while chitosan is valued for its biocompatibility, film-forming ability, and antimicrobial activity, it is limited by poor solubility at neutral pH; alginate, though widely used for its gelling and moisturizing properties, lacks intrinsic bioactivities that target skin aging pathways.^26–28^ In contrast, fucoidan combines favorable formulation properties with multifunctional bioactivity, positioning it as a unique candidate among marine polysaccharides for skincare applications.

To maximize the therapeutic potential of fucoidan and other marine-derived bioactives, liposomal encapsulation offers a sophisticated delivery strategy. Liposomes are nanoscale vesicles composed of lipid bilayers that can carry both hydrophilic and lipophilic compounds, thereby enhancing dermal penetration while protecting bioactive compounds from oxidative degradation.^29^ Algae lipids, such as palmitic and oleic acids, provide a biocompatible and sustainable matrix that improves liposome stability and additionally confers skin benefits, including barrier reinforcement, wrinkle reduction, and mitigation of acne-related efflorescence.^30,31^ In recent years, algal-based nanocarriers have demonstrated improved photostability and antioxidant retention, particularly in skincare applications.^26,32,33^ The novelty of the present approach lies in the dual-functional design of liposomes derived from *Sargassum* lipids and coated with fucoidan, wherein both the carrier and the encapsulated compound contribute to the bioactivity. This complementary configuration not only enhances delivery efficiency but also establishes an environmentally sustainable route for valorizing *Sargassum* biomass into high-value skincare products within a circular economy framework.^34^

Recent advances in polysaccharide-coated nanocarriers underscore the potential of surface functionalization to enhance liposomal stability, bioactivity, and applicability in both biomedical and cosmetic applications. Although fucoidan-coated liposomes have been primarily explored in pharmaceutical formulations, their translation into cosmeceutical platforms remains limited, in part due to challenges related to stability under the environmental stress of sustainable raw materials. To address these gaps, this study introduces a novel dual-valorization strategy combining *Sargassum*-derived lipids with a fucoidan coating, thereby transforming an environmental liability into a functional resource for skincare innovation (Figure 1). This work aims to bridge marine biomass valorization with advanced delivery systems, serving as a critical foundation for subsequent biological and dermatological testing. The goal is to generate quantitative data supporting the safety and efficacy of the carrier system, informing future in vivo studies and product development in alignment with regulatory standards.

**Figure 1:**
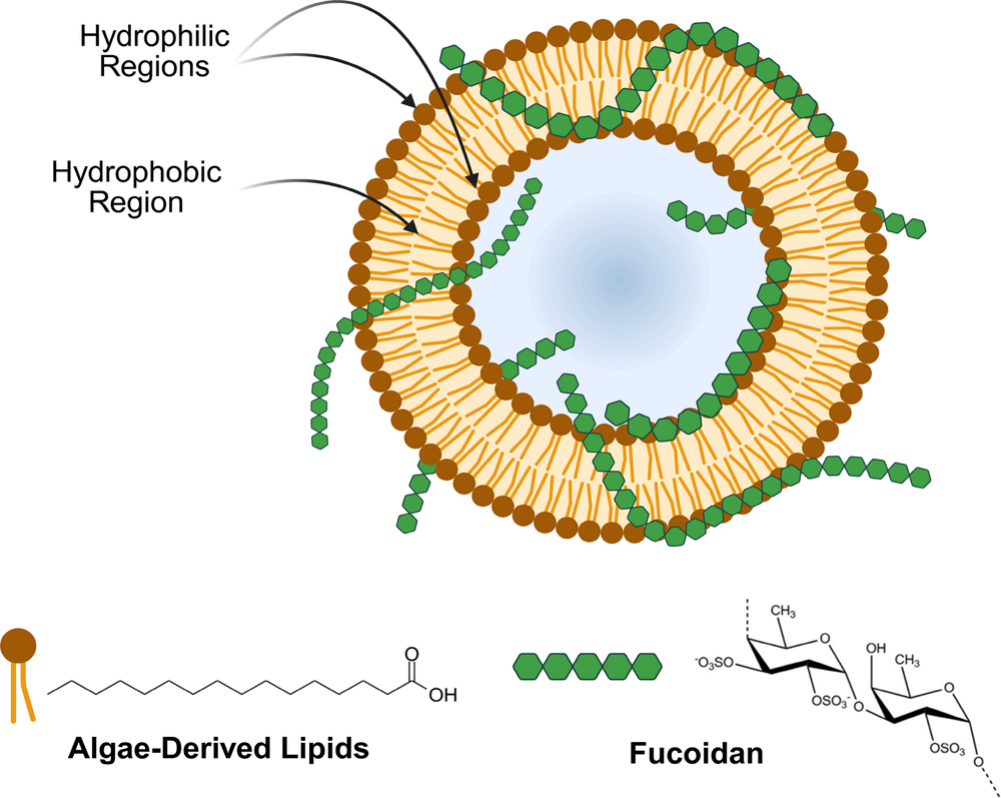
Schematic representation of fucoidan-coated liposomes, including algae-derived lipids such as palmitic acid and fucoidan from *Sargassum*, providing physical and photostability (image created in BioRender).

We report the synthesis and characterization of these marine-derived liposomes, assessing key physicochemical properties such as particle size, polydispersity index (PDI), and zeta potential along with their thermal behavior and molecular composition through Fourier-transform infrared spectroscopy (FTIR), gas chromatography (GC), differential scanning calorimetry (DSC), and thermogravimetric analysis (TGA). Accordingly, the present work quantitatively evaluates the physicochemical stability and antioxidant performance of fucoidan-coated *Sargassum* liposomes to establish their potential as sustainable skincare nanocarriers. By examining the role of fucoidan in enhancing liposomal stability and photoprotection, this work aims to provide new insights into the development of multifunctional, sustainable nanocarriers that can mitigate oxidative stress and UV-induced skin damage.

Fucoidan-coated *Sargassum* liposomes represent a versatile, sustainable, and eco-friendly delivery platform with significant potential for use in skin care products, skin therapy, and the delivery of unstable or labile drugs. Liposomal systems are widely recognized for their ability to improve the stability, bioavailability, and targeted delivery of chemotherapeutic agents, reducing systemic toxicity and enhancing therapeutic efficacy.^35^ Additionally, their protective properties make them suitable for encapsulating sensitive compounds such as antioxidants, anti-inflammatory agents, and UV filters in skincare formulations.^36^ These features, combined with the bioactivity of fucoidan, position them as promising carriers for both therapeutic and cosmetic applications within a circular economy framework.

## 2 Materials and Methods

### 2.1 Reagents

Hexane (GC grade), chloroform (HPLC grade), methanol (HPLC grade), ethanol, sodium hydroxide (NaOH), hydrochloric acid (HCl), sephadex G50 (fine), fucoidan (Fucus vesiculosus, ≥95%), 2,2′-azino-bis­(3-ethylbenzothiazoline-6-sulfonic acid) diammonium salt (ABTS), and potassium persulfate were purchased from Sigma-Aldrich. Pure nitrogen was purchased from Messer.

### 2.2 Materials

*Sargassum* samples were collected in Palmas del Mar Beach, Humacao, Puerto Rico, in April 2024. The biomass was washed thoroughly with distilled water to remove any sand, shells, or salt; the pellet was then lyophilized. The dried biomass was ground and screened to a size smaller than 100 μm using a 150 mesh screen. The ground *Sargassum* was stored in a −20 °C freezer until further analysis.

### 2.3 Lipid Extraction and Characterization

The extraction method was adapted from Jeong et al.^37^ Extractions were performed in triplicate (*n* = 3). Approximately 500 mg of dry powder and 10 mL of chloroform:methanol (2:1, v/v; Folch method) were mixed in an extraction container and mixed at 500 rpm for 2 h at 45 °C. The extract was then separated by centrifugation for 10 min at 4,000 rpm at 45 °C. Higher temperatures and times enhance solvent dissolution capacity, solubility, and diffusion rates but also incur higher operational costs, making 45 °C and 2 h optimal. The solvent was evaporated (rotary evaporator, bath ≤ 40 °C), and the supernatant was weighed; the lipid yield was calculated as

$$\mathrm{Lipid}\;\mathrm{Yield}\;(\%)=\frac{\mathrm{mass}\;\mathrm{of}\;\mathrm{dried}\;\mathrm{lipid}\;\mathrm{residue}\;(\mathrm{mg})}{\mathrm{mass}\;\mathrm{of}\;\mathrm{dry}\;\mathrm{sample}\;(\mathrm{mg})}\times 100$$

### 2.4 Preparation of *Sargassum*-Based Liposomes

This method was modified from Tziveleka et al.^38^ Different liposomal formulations were prepared using the thin-film hydration method and algal-derived lipids with a reported composition of polyunsaturated, monounsaturated, and saturated fatty acids. Lipids (10 mg/mL) were dried overnight under vacuum to remove residual solvent and then hydrated with 1 mg/mL fucoidan solutions in nanopure water, a concentration optimized based on literature reports demonstrating effective cellular activity without significant cytotoxicity.^39,40^ The lipid-to-aqueous phase ratio was 1:1 (v/v). Hydration was performed by gentle stirring for 1 h in a water bath at a temperature above the main transition temperature (Tm) to facilitate bilayer formation (>45 °C). A Branson 2510 ultrasonic cleaner processed the liposome suspensions with a frequency of 40 kHz and power density of 36 W/L (Branson Ultrasonics, Danbury, CT, USA). Since amplitude is not user-adjustable in bath sonicators, energy delivery is defined by this fixed frequency and power density. Samples were placed in the bath without contact with the tank bottom and sonicated for two 3 min cycles, separated by 3 min cooling intervals. Indirect bath sonication was selected to minimize the risk of metal contamination associated with direct probe sonication. The vesicles were allowed to rest for 30 min and then separated using Sephadex G50 columns.

### 2.5 Physicochemical Characterization of Liposomes

Empty and fucoidan-loaded liposomal systems were physicochemically characterized using dynamic light scattering (DLS) methods to determine their size and stability, as measured by a Zetasizer Pro instrument (Malvern Instruments, U.K.). Samples (200 μL) were diluted in 3 mL of nanopure water (refractive index 1.45 at 25 °C) and equilibrated at 25 °C in a disposable polystyrene cuvette. Hydrodynamic diameter (Dh), polydispersity index (PDI), and ζ-potential were measured at 173° backscatter and 633 nm, with automatic attenuator settings. Each measurement consisted of 100 runs and was performed in triplicate (*n* = 3). Analyses were conducted immediately after production (*t* = 0) and after storage at 4 °C for 28 days to assess physical stability.

### 2.6 Photodegradation of Liposomes

The photodegradation of liposomes was tested by using a 75 W xenon arc lamp (Model 6251NS, Newport Corporation, USA). The lamp emitted continuously from 200 to 2500 nm, with strong output in the 200–800 nm UV–vis region. Samples (5 mL) were placed 0.5 m from the lamp housing, giving an irradiance of ∼0.20–0.25 mW cm^–2^ in the UV–vis range. Irradiation was performed in quartz cuvettes with an optical path length of 1 cm. Cuvettes were oriented perpendicular to the collimated beam for a uniform exposure. Samples were stirred at 100 rpm for 30 min at 25 °C. All experiments were run in triplicate (*n* = 3).

### 2.7 Fourier-Transform Infrared Spectroscopy

FTIR spectra of the lyophilized *Sargassum*-based liposomes were analyzed at 0 and 30 min of photodegradation using a Spectrum Two FTIR Spectrometer (PerkinElmer, USA) coupled with an attenuated total reflectance (ATR) cell. The sample was pressed onto the crystal with a controlled contact pressure of ∼80 N using the built-in pressure tower. Spectra were acquired at room temperature and averaged over the 400–400 cm^–1^ range with 16 scans and a resolution of 1 cm^–1^. An air background was subtracted before each sample, and the crystal was cleaned before each run. All measures were performed in triplicate (*n* = 3); replicate spectra were overlaid, and the mean ± SD was used for analysis to confirm fucoidan signature peaks and assess molecular degradation/compositional changes.

### 2.8 Gas Chromatography

Fatty acid composition of macroalgae lipid extracts was determined via saponification, methylation, and hexane extraction, followed by GC-MS analysis. The extraction protocol was adapted from a bacterial protocol reported by Sasser et al.^41^ Aliquots of 3 mL from 20 mL of lipid extracts were taken for characterization. Saponification was performed by adding 3 mL of 4 M NaOH in a methanol:water (1:1) mixture, heating at 80 °C for 1 h, and then storing at 4 °C. For methylation, 5 mL of 4 M HCl in methanol was added and heated at 80 °C for 1 h. Methylated fatty acids were extracted with 10 mL of hexane and collected in the hexane phase. Nitrogen was used to evaporate the solvent to 1 mL.

For GC-MS analysis, an Agilent 7890A GC was coupled with an Agilent 5975C inert XL MSD. An autosampler injects 1 μL of the sample, with the inlet set to splitless mode at 250 °C, a temperature ramp of 6 °C/min from 50 to 300 °C, and an HP-5MS column (30 m × 320 μm × 0.25 μm). Only mass spectra peaks corresponding to chemical compounds with a match of greater than 70% to the NIST library were considered for further characterization and classification. Measurements were performed in triplicate; solvent and method blanks were analyzed to confirm the absence of contamination. This method was applied to characterize lipid profiles of macroalgae-based liposomes and to monitor compositional changes before and after photodegradation experiments.

### 2.9 Differential Scanning Calorimetry

The thermal behaviors of lyophilized empty and fucoidan-coated liposomes were analyzed using a Mettler Toledo TGA-DSC 3+ Thermal Analysis System. Approximately 3 mg of the samples were placed in sealed aluminum pans, with an empty pan as a baseline. The analysis involved heating the samples from room temperature to 300 °C at a controlled rate of 10 °C/min with dry nitrogen used as the purge gas at a flow rate of 60 mL/min. This thermal analysis aimed to evaluate the stability and thermal behavior of the liposomes under extreme temperature conditions, both before and after the photodegradation process.^42^

### 2.10 Thermal Gravimetric Analysis

The TGA of the lyophilized liposome samples was performed using a Mettler Toledo TGA-DSC 3+ Thermal Analysis System. Approximately 3 mg of the samples were heated from room temperature to 300 °C at a rate of 10 °C/min under a dry nitrogen atmosphere.

### 2.11 ABTS Antioxidant Activity

The antioxidant scavenging activity was evaluated against the 2,2′-azino-bis­(3-ethylbenzothiazoline-6-sulfonic acid) radical cation (ABTS•+).^43^ The ABTS radical solution (3.5 mmol L^–1^) will be prepared by mixing 5 mL of ABTS stock solution (7 mmol L^–1^ in water) with 5 mL of potassium persulfate, K2S2O8 (2.45 mmol L^–1^ in water). This mixture was kept for 12–16 h in the dark at room temperature. It was diluted in ethanol to obtain an absorbance value of ≈ 0.9 measured at 734 nm using a 200 ProTecan Plate Reader. The samples (0.13–2 mg/mL in ethanol) were combined with the ABTS solution by adding 75 μL of the sample or pure solvent and 75 μL of the diluted ABTS•+ solution in a 96-well transparent plate in triplicate. The plate was incubated in the dark at 30 °C for 30 min, and then the absorbance was measured at 734 nm. All measurements were performed in triplicate (*n* = 3). Negative controls included water and ethanol. The positive control is ascorbic acid. The % of ABTS radical remaining can be determined according to the equation:

$$\mathrm{Scavenging}\;(\%)=\frac{A_{\mathrm{control}}-A_{\mathrm{sample}}}{A_{\mathrm{control}}}\times 100$$

where *A*_control_ is the absorbance at 734 nm of the radical ABTS in the solvent, and *A*_sample_ is the absorbance at 734 nm of the radical ABTS in the sample. To determine the IC50, which defines the concentration of antioxidants required to achieve a 50% scavenging efficiency, a linear regression analysis was performed using the concentrations of samples and the percentage of the inhibition curve. The activity, expressed as ascorbic acid equivalents (AAEe) using the equation:

$$\mathrm{AEE}\;\left(\frac{\mu \mathrm{mol}}{g}\right)=\frac{{\mathrm{IC}}_{50}\mathrm{AA}\;\left(\frac{\mu \mathrm{mol}}{L}\right)}{{\mathrm{IC}}_{50}\;\mathrm{sample}\;\left(\frac{g}{L}\right)}$$

### 2.12 Statistical Analysis

All statistical analyses were conducted using OriginPro version 9.0.0. (Northampton, MA, USA). Two-group comparisons were performed using paired Student’s *t* tests. To compare the mean values among groups, one-way or two-way analysis of variance (ANOVA) was used, followed by Tukey’s post hoc test. Data were expressed as mean ± SD. All p values less than 0.05 were considered statistically significant.

## 3 Results and Discussion

### 3.1 Yield Percentage in *Sargassum* Extraction

Extraction of total lipids from pelagic *Sargassum* with chloroform:methanol solvent (2:1, v/v) resulted in a lipid content of 3.86% dry weight, showing this solvent mixture’s effectiveness for retrieving bioactive lipid fractions. Jeong et al. demonstrated the efficiency of this method, finding that among 13 tested solvents, the chloroform:methanol (2:1) mixture yielded the highest lipid content from *Enteromorpha intestinalis* due to its intermediate polarity. This solvent system facilitates the extraction of both neutral and polar lipids, many of which have skin-beneficial properties.^37^ The 3.86% yield obtained in this study suggests that *Sargassum* contains a relatively high lipid content, potentially rich in fatty acids and other compounds that could be beneficial in skincare formulations. These findings support the value of *Sargassum*-derived lipids as sustainable ingredients for skincare products.

### 3.2 Particle Size, PDI, and Zeta Potential

The physicochemical traits of empty and fucoidan-coated liposomes are summarized in Table 1. Empty liposomes exhibited an average particle size of 700 nm, while fucoidan-coated liposomes showed a modest reduction to 670 nm. The overall particle size range is consistent with vesicles prepared by thin-film hydration, which predominantly yield multilamellar vesicles (MLVs) larger than 500 nm. Zeta potential measurements revealed that empty liposomes carried a charge of −36 mV, indicating a stable formulation, while fucoidan-coated liposomes exhibited a significantly higher negative potential of −72 mV.

**Table 1:** Physicochemical Changes in Empty and Fucoidan-Coated Liposomes during Storage at 4°C^a^

formulation	day	Dh (nm)	PDI	ζ (mV)
empty liposomes	0	700 ± 10	0.8 ± 0.3	–36.8 ± 0.2
empty liposomes	7	740 ± 60	1.0 ± 0.1	–36 ± 1
empty liposomes	14	540.4 ± 0.5	0.6 ± 0.4	–39 ± 1
empty liposomes	28	600 ± 10	0.7 ± 0.6	–36 ± 4
fucoidan-coated liposomes	0	673 ± 60	0.8 ± 0.3	–72 ± 8
fucoidan-coated liposomes	7	600 ± 10	0.6 ± 0.4	–72 ± 3
fucoidan-coated liposomes	14	461.8 ± 30	0.2 ± 0.1	–85 ± 7
fucoidan-coated liposomes	28	540 ± 20	0.5 ± 0.4	–47 ± 3

a Both show good stability as liposomes, but adding fucoidan enhances stability while interacting with the lipid bilayer.

Particle size and zeta potential are critical determinants of liposomal stability, distribution, and biological performance. Small vesicles provide a larger surface-to-volume ratio, enhancing solubility, release properties, and targeting accuracy.^44,45^ The relatively large particle sizes observed here are characteristic of MLVs formed by thin-film hydration. The slight size reduction observed upon fucoidan coating is likely due to electrostatic interactions between fucoidan, a negatively charged polysaccharide, and the lipid bilayer. Such interactions may promote tighter bilayer packing; however, the low fucoidan concentration resulted in a heterogeneous size distribution and weaker interparticle repulsion.

Zeta potential further supports these findings, as values above ±30 mV are considered stable due to strong interparticle electrostatic repulsion, thereby preventing coagulation. Conversely, lower values lead to particle aggregation.^46^ Empty liposomes (−36 mV) already demonstrated stability, but fucoidan coating significantly enhanced this effect; the potential nearly doubled to −72 mV. This pronounced negative charge indicates robust electrostatic stabilization, reducing the likelihood of aggregation or precipitation. Notably, the combination of modest size reduction and a substantial increase in surface charge suggests that fucoidan not only contributes to structural modification but also enhances the overall physicochemical stability of the liposomes, which is expected to improve circulation time and therapeutic performance.

The physical stability of liposomes was assessed during storage at 4 °C for 28 days (Table 1 and Figure 2). Empty liposomes (EMPL) remained large and heterogeneous throughout storage (Dh 700 → 740 → 540.4 → 600 nm; PDI 0.8 → 1.0 → 0.6 → 0.7 from 0 to 28 days), with the day-7 increase in size and PDI suggesting aggregation and a multimodal size distribution. In contrast, FDL contracted and sharpened by day 14 (Dh 673 → 600 → 461.8 → 540 nm; PDI 0.8 → 0.6 → 0.2 → 0.5), accompanied by strongly negative ζ-potentials (−72 → −72 → −85 → −47 mV). On day 14, the combination of smaller Dh, low PDI (∼0.2), and ζ ≪ −30 mV indicated effective electrostatic stabilization from the anionic polysaccharide coating.

**Figure 2:**
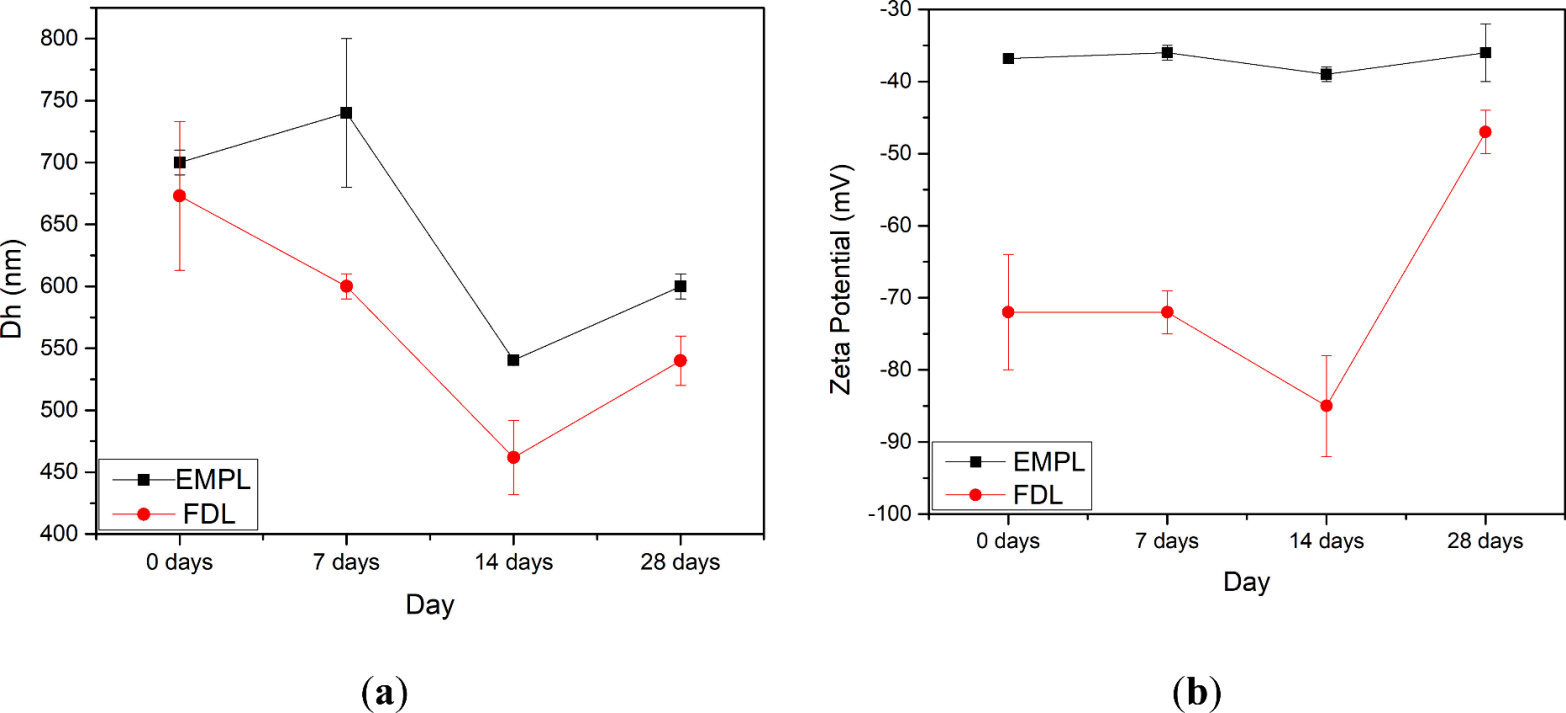
Line plots of (a) particle size and (b) zeta potential changes of empty and fucoidan-coated liposomes for 28 days. These physicochemical properties demonstrate the physical stability of fucoidan-coated liposomes during storage with a lower particle size and high stability.

Although a partial rebound was observed at 28 days (Dh 540 nm; PDI 0.5; ζ −47 mV), this is more likely due to aging-related effects, such as counterion screening, partial desorption of fucoidan, or bilayer rearrangements, rather than an intrinsic failure of the coating. Overall, fucoidan coating significantly enhanced colloidal stability compared with uncoated vesicles, particularly over the first 2 weeks of storage. From a formulation perspective, these findings support the adoption of fucoidan-coated liposomes as a baseline design while emphasizing the need for optimization strategies, such as increasing coating density, improving anchoring interactions, adjusting ionic strength and pH, or adding a steric stabilizer, to achieve stability beyond 2 weeks. Setting predefined acceptance criteria (e.g., PDI ≤ 0.3–0.5, ζ ≥ 30–40 mV) will further ensure consistency and robustness for long-term storage.

### 3.3 Fourier-Transform Infrared Spectrometer

FTIR spectral analysis was conducted to confirm the incorporation of fucoidan into liposomal formulations (Figure 3). Empty liposomes exhibited characteristic peaks of fatty acids at wavenumbers of 3353 cm^–1^ (OH linkage between water and algal lipids), 2919 cm^–1^ (CH_2_ asymmetric stretching vibration), 2849 cm^–1^ (CH_2_ symmetric stretching vibration), and 1735 cm^–1^ (symmetric stretching vibration of C=O groups), which are typical of lipid bilayers.^47^ Fucoidan-coated liposomes exhibited a broader peak of 3367 cm^–1^ (OH stretching), which belongs to fucoidan’s intramolecular or intermolecular hydrogen bonds. Additional peaks at 1621 cm^–1^ (acyl group), 1218 cm^–1^ (sulfate group), 1019 cm^–1^ (C–O–C and C–O–H stretching), and 816 cm^–1^ (β-glycosidic bonds between the monosaccharide units) were observed.^48^ These peaks, absent in empty liposomes, are characteristic of fucoidan and confirm its successful incorporation into the liposomal structure.

**Figure 3:**
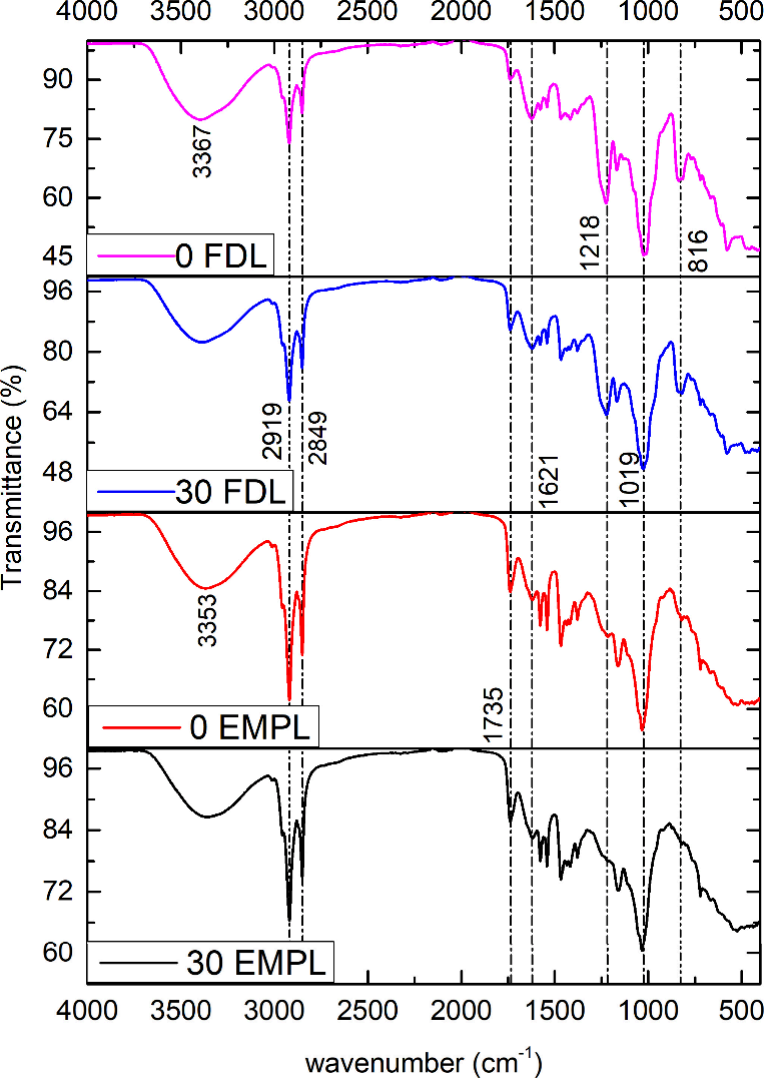
FTIR spectra of empty (EMPL) and fucoidan-coated (FDL) liposomes (1 mg/mL fucoidan) before and after photodegradation with a 75 W xenon arc lamp. No significant changes were shown in their characteristic bands, showing good stability for skincare applications.

These results are consistent with previous reports on fucoidan-coated liposomes, where sulfate- and glycosidic-related vibrations served as diagnostic markers. The slight shift of the OH band likely reflects differences in fucoidan origin or liposomal composition, which suggests a specific hydrogen bonding interaction with lipid head groups.^49^

In addition to confirming incorporation, the FTIR spectra reveal how the molecular organization of the fucoidan-lipid complex responds to experimental conditions. The minor yet consistent shifts in the OH and C = O stretching regions suggest a subtle reorganization of hydrogen bonding networks between fucoidan sulfate groups and lipid head groups. Upon irradiation, decreases in transmittance around 1200–800 cm^–1^ correspond to the cleavage of glycosidic bonds and potential formation of new COOC groups near 1735 cm^–1^, indicating partial oxidation or rearrangement of polysaccharide chains. These spectral modifications are consistent with previously reported FTIR observations of structural changes in fucoidan and other sulfated polysaccharides upon photochemical or enzymatic treatment, where variations in sulfate and glycosidic bond intensities reflect complex formation and degradation pathways.^50,51^ Similar FTIR shifts related to sulfate and hydrogen bonding interactions have been described in fucoidan-liposome systems, supporting the notion that formulation variables can influence intermolecular organization and stability.^52,53^ Such evidence suggests that the kinetics of complex formation and degradation are modulated by both molecular interactions and environmental exposure, highlighting the importance of controlling the formulation parameters to preserve the structural integrity and functionality.

Collectively, these spectral observations not only confirm the successful coating of liposomes with fucoidan but also clarify how their chemical interactions evolve under the processing and photodegradation conditions. Such molecular interactions may enhance stability and functional performance, supporting their potential in skincare applications.

Fucoidan-loaded samples may exhibit changes after photodegradation and subsequent irradiation. Analysis through FTIR helps identify changes in fucoidan’s characteristic bands or the emergence of new peaks; this supports the development of a stable, sustainable skincare alternative. This approach enhances the stability of macroalgae-derived compounds, ensuring optimal protection during delivery and transportation. FTIR spectra analysis, as shown in Figure 3, revealed statistically significant differences in transmittance between 0 and 30 min of degradation at all the characteristic peaks (*p* < 0.05). However, the observed mean (Table 2) differences were minor, with changes corresponding to less than 5% of the initial signal. While the statistical tests confirm that the variation is unlikely due to random error, the magnitude of the change is within the expected experimental variation. It does not indicate substantial chemical degradation, indicating their potential suitability in skincare formulations.^54^ A decrease in transmittance after irradiation in the regions of 1200 and 800 cm^–1^ can confirm the break of glycosidic bonds and the formation of COOC groups, as shown in the peak at 1738 cm^–1^. OH bands also show a change in transmittance, which can be due to the lyophilization process used for the samples, as water can bind to the algal lipids.

**Table 2:** Means of Different FTIR Peak Positions of Characteristic Functional Groups in Liposomal Formulations^a^

peak (cm^–1^)	time (min)	mean ± SD (%)	Δ Difference
2919 (EMPL)	0	62.4 ± 0.9	3.91
	30	66.36 ± 0.08	
2849 (EMPL)	0	71.7 ± 0.3	3.27
	30	74.9 ± 0.5	
2919 (FDL)	0	67.09 ± 0.01	4.41
	30	71.5 ± 0.8	
2849 (FDL)	0	77.4 ± 0.8	4.56
	30	81.9 ± 0.5	
1216 (FDL)	0	62.8 ± 0.7	3.74
	30	59.1 ± 0.1	
816 (FDL)	0	68.9 ± 0.2	4.17
	30	64.78 ± 0.05	

a All changes are statistically significant (*p* < 0.05); however, they have less than a 5% impact on ensuring structural integrity.

### 3.4 Gas Chromatography

GC-MS analysis was carried out to characterize the lipid composition and assess the photostability of both empty and fucoidan-coated liposomes (Figure 4). Both formulations were found to be rich in polyunsaturated (PUFAs), monounsaturated (MUFAs), and saturated fatty acids, collectively accounting for over 50% of the detected peaks. The results (Figure 4a) showed the presence of three prominent compounds: hexadecenoic acid (palmitic acid (PA); C16:0), 5,8,11,14-eicosatetraenoic acid methyl ester (all-Z) (arachidonic acid (AA); C20:4­(ω-6)), and 9-octadecenoic acid (oleic acid (OA); C18:1­(ω-9)). These fatty acids represent saturated, polyunsaturated, and monounsaturated lipid species, each contributing uniquely to the structural and functional characteristics of the lipid matrix.

**Figure 4:**
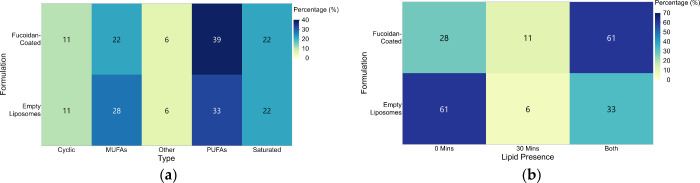
Heat maps of GC-MS data that include (a) fatty acid composition in empty and fucoidan-coated liposomes, showing differences in PUFAs, MUFAs, saturated, cyclic, and other lipid types, in which fucoidan-coated liposomes exhibited a higher PUFA content; and (b) lipid detection over time under photodegradation stress. Empty liposomes showed greater degradation, while fucoidan-coated liposomes retained more lipids at both time points, indicating enhanced stability.

Under photodegradation stress (Figure 4b), distinct compositional differences were observed between the two formulations. For empty liposomes, 61% of lipid species were detected only at 0 min, and 33% persisted after 30 min of UV exposure, which suggests significant lipid degradation. In contrast, fucoidan-coated liposomes retained 61% of lipids across both time points, indicating improved resistance to photodegradation, although 28% was lost after irradiation. The enhanced photostability observed in fucoidan-coated liposomes can be attributed to the dual protective mechanism of fucoidan. The polysaccharide layer acts as a physical barrier, limiting direct UV exposure and protecting the lipid bilayer from oxidative stress.^55^ At the same time, its antioxidant capacity scavenges reactive oxygen species that promote lipid peroxidation. This dual function aligns with previous reports describing fucoidan’s radical scavenging activity and membrane-stabilizing effects in nanocarriers. Similar stabilization has been observed in chitosan-coated liposomes, where the polysaccharides enhance lipid membrane integrity through surface protection and electrostatic stabilization.^56,57^ The partial degradation still observed after 30 min indicates that, although fucoidan substantially slows oxidative reactions, secondary degradation pathways may persist within the bilayer.

The observed lipid composition validates the skincare relevance of the formulations. In the context of skincare, PA is known for its ability to enhance the skin barrier function and emollient properties.^58,59^ AA is a key omega-6 fatty acid that plays a role in modulating inflammatory responses and may support skin repair and regeneration.^60,61^ OA is an omega-9 fatty acid with properties that enhance penetration but may induce irritation when applied alone.^62^ Encapsulation within liposomes mitigates these drawbacks by regulating lipid release and improving biocompatibility, as demonstrated in similar liposomal systems containing essential fatty acids.^63^

The FAME compositions of both liposomal systems were further analyzed before and after photodegradation (Figure 5). Across all samples, hexadecenoic acid (PA) was the predominant compound, representing 55–65% of the total composition. Minor fractions of methyl tetradecanoate (C14:0) and methyl stearate (C18:0) were detected alongside significant levels of polyunsaturated methyl esters, such as 5,8,11,14-eicosatetraenoic acid methyl ester. After 30 min of UV exposure, empty liposomes exhibited pronounced compositional shifts, particularly in polyunsaturated FAMEs, indicative of early oxidative degradation. In contrast, the fucoidan-coated liposomes maintained a largely preserved FAME profile. This confirms their superior resistance to photodegradation.

**Figure 5:**
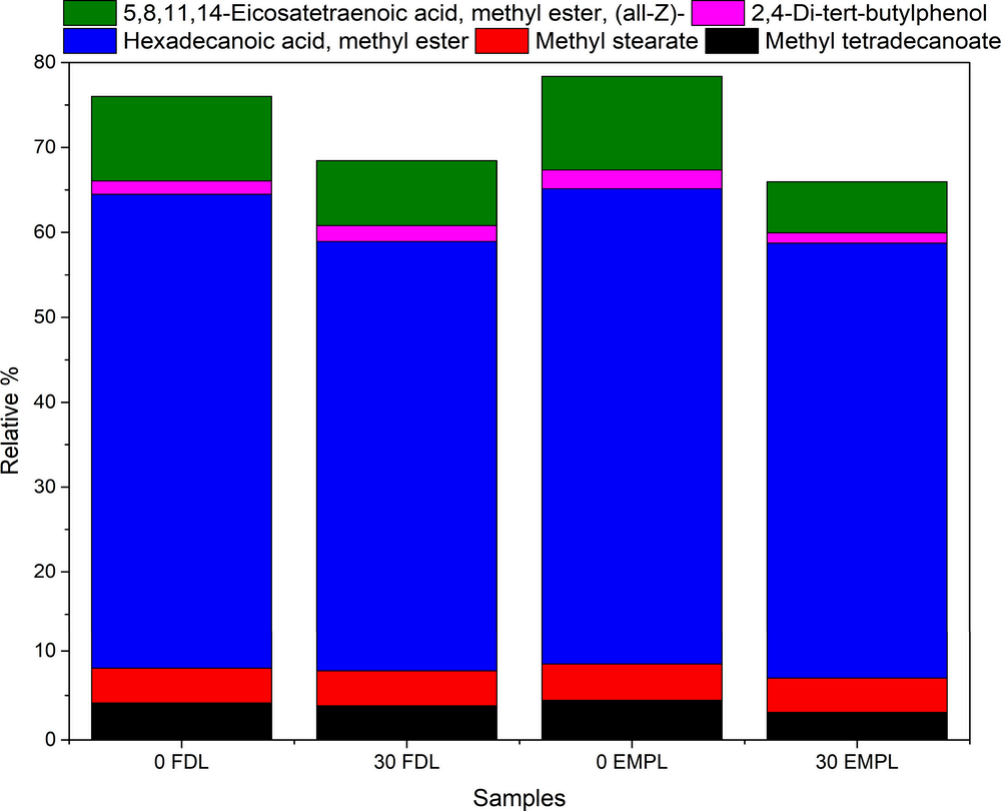
Relative composition (%) of fatty acid methyl esters (FAMEs) in fucoidan-coated (FDL) and uncoated (EMPL) samples at 0 and 30 min, showing greater stability of FAMEs with fucoidan coating. The observed changes were statistically significant (*p* < 0.05) yet contributed to less than 5% variation, thereby preserving structural integrity.

This preservation can be attributed to fucoidan’s instantaneous barrier function, which limits oxygen diffusion and contact between reactive species and the lipid matrix, as well as its radical scavenging activity, which suppresses peroxidation of unsaturated esters. After 30 min, the partial retention of polyunsaturated esters highlights fucoidan’s fast-acting stabilization capability, a critical property for lipid systems prone to early-stage oxidation. Statistical evaluation using one-way ANOVA tests confirmed statistically significant differences (*p* < 0.05) between the FAME composition of empty and fucoidan-coated liposomes. Collectively, these findings support the conclusion that fucoidan serves as an effective natural stabilizer, mitigating rapid lipid oxidation and enhancing the photochemical durability of liposomal formulations. These analyses highlight fucoidan’s potential as a biocompatible coating material for developing photostable, antioxidant-enriched liposomes for skincare applications.

### 3.5 Differential Scanning Calorimetry

DSC studies (Figure 6) were conducted to examine the thermal transitions and stability of the liposomal systems before and after photodegradation. The untreated liposomes exhibited an endothermic peak near 50 °C, indicating the liposomal lipids’ gel-to-liquid crystalline phase transition (*T*_m_). The sharp peak indicates a homogeneous lipid environment, whereas the broadened and shifted peak in fucoidan-coated liposomes reflects polymer–lipid interactions that alter bilayer packing fluidity.

**Figure 6:**
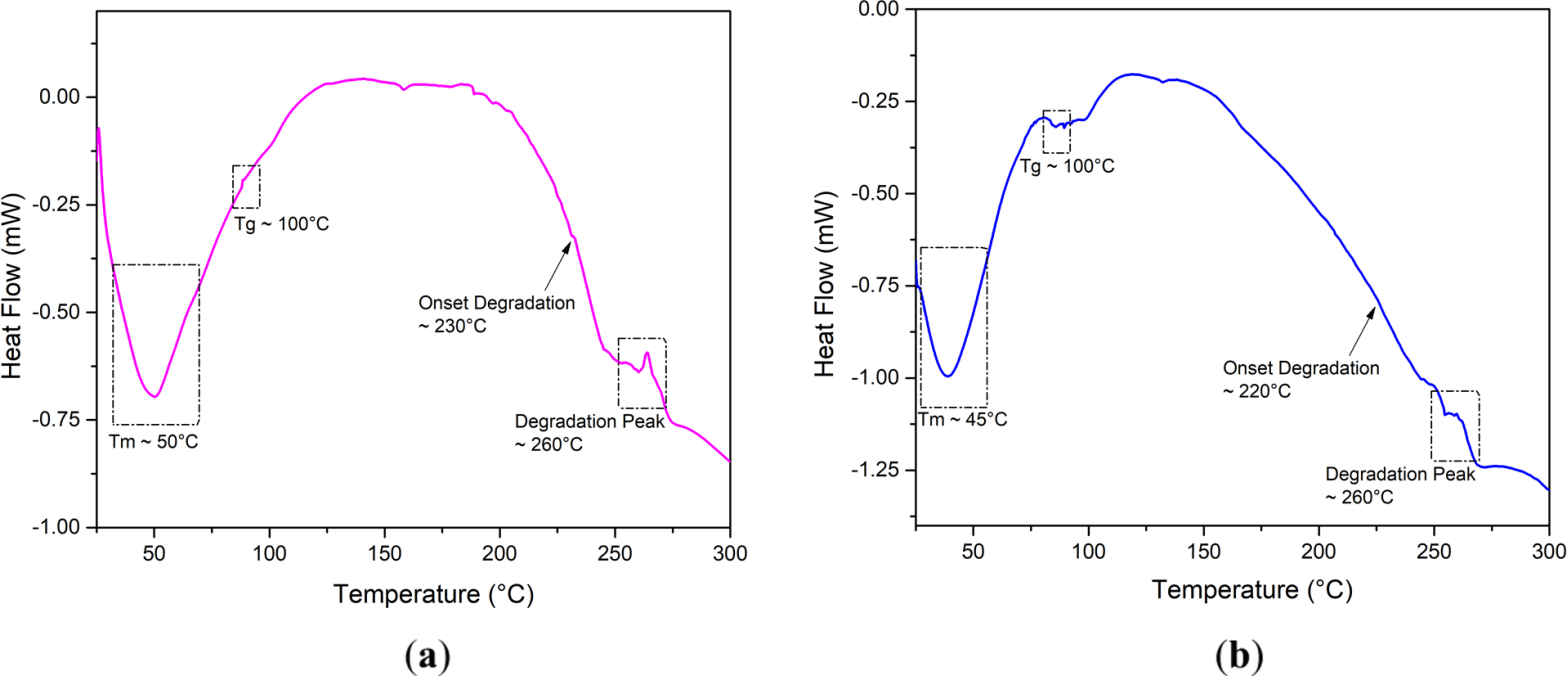
DSC thermogram of fucoidan-coated liposomes (1 mg/mL fucoidan) before (a) and after (b) photodegradation from 25 to 300 °C. It demonstrates the effect of fucoidan on the thermal behavior of liposomal drug delivery.

After photodegradation, liposomes exhibit increased bilayer disorder, which can be attributed to lipid peroxidation or partial membrane destabilization, such as that resulting from photo-oxidative stress.^64^ A second endothermic event around 260 °C corresponded to the thermal degradation or decomposition of fucoidan’s glycosidic bonds or the oxidation of lipid chains.^65^ Followed by a downward trend beyond 270 °C, indicating fucoidan carbonization, a typical phenomenon for polysaccharide-based systems. The photodegraded samples exhibited broader and more intense peaks, suggesting a reduced overall thermal stability.

Fucoidan enhanced stability in the intermediate temperature range (150–250 °C) by forming hydrogen bonds and electrostatic interactions with lipid head groups, thereby strengthening bilayer cohesion. These results confirm that fucoidan acts as a thermal protective coating, improving liposome stability, and supporting the controlled release of encapsulated compounds; however, light exposure still decreases thermal resilience due to oxidative degradation.

### 3.6 Thermal Gravimetric Analysis

TGA was employed to evaluate the thermal stability of fucoidan-coated liposomes before and after photodegradation, and any mass change during this process was recorded (Figure 7). The thermograms revealed a multistep degradation profile that reflects the interactions between the lipid bilayer and fucoidan. The initial weight loss (9%) below 100 °C corresponds to the evaporation of surface-bound or loosely associated moisture, suggesting that most of the remaining water is hydrogen-bonded or retained within the vesicle structure. The significant weight loss (30%) occurred around 230 °C, representing the primary thermal degradation phase associated with fucoidan backbone decomposition and lipid oxidation. This degradation at this temperature is consistent with the known behaviors of sulfated polysaccharides (via depolymerization and decomposition) and phospholipid assemblies under oxidative thermal stress. After 250 °C, carbon combustion occurs.^66^

**Figure 7:**
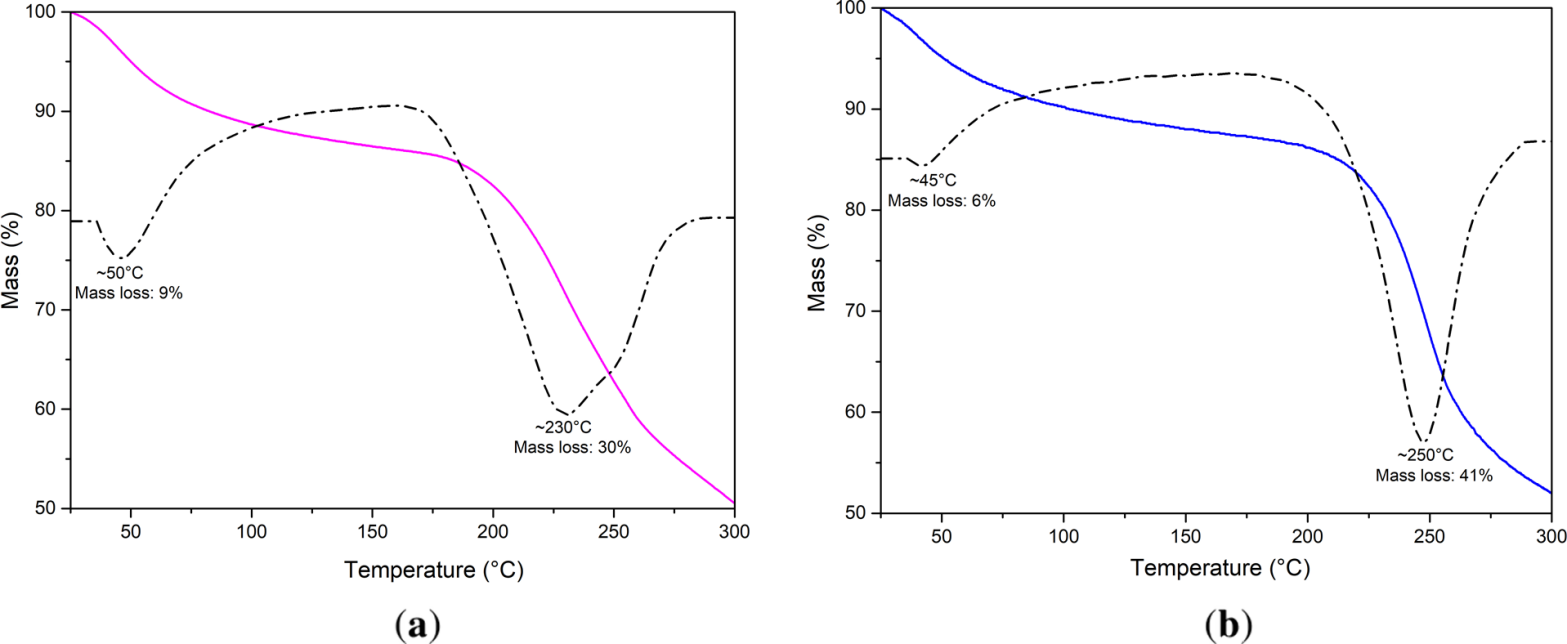
TGA of fucoidan-coated liposomes (1 mg/mL fucoidan) before (a) and after (b) photodegradation from 25 to 300 °C. The superior thermal stability of photodegraded fucoidan-coated liposomes is attributed to the protective effect of the liposomes upon UV irradiation.

The thermograms of the photodegraded samples showed notable differences from those of the untreated systems. A lower mass loss (6%) indicates reduced moisture retention, likely due to partial bilayer disruption or conformational rearrangements in the fucoidan coating after UV exposure. However, the curves show higher stability between 90 and 180 °C, implying that partial cross-linking or oxidative modification of fucoidan may enhance intermediate-range thermal resistance. The main degradation phase (41%) shifted toward 250 °C and became more abrupt, suggesting decomposition of prefragmented lipid or fucoidan components generated during photodegradation. Despite these changes, the fucoidan-coated liposomes maintained structural integrity through most of the heating range, confirming fucoidan’s protective stabilizing role.

Like DSC results, TGA results indicate that fucoidan-coated liposomes exhibit improved thermal stability and retain cohesive structure even after UV exposure. The slight enhancement in stability following photodegradation may result from polymer cross-linking or reorganization of fucoidan chains, which confers resistance to further thermal oxidation. These findings reinforce fucoidan’s potential as a thermally stable, photoresistant coating suitable for skincare applications.

### 3.7 ABTS Antioxidant Activity

Both empty and fucoidan-loaded liposomes were analyzed before and after exposure to light to evaluate the antioxidant activity of liposomal encapsulation and the impact of photodegradation (Figure 8). The antioxidant activity of empty and fucoidan-loaded liposomes was assessed using the 2,2′-azino-bis­(3-ethylbenzothiazoline-6-sulfonic acid) radical cation assay (ABTS), which measured the free radical scavenging capacity of both encapsulations. Ascorbic acid was used as the positive control in all tests. A dose-dependent increase was observed in all samples before and after photodegradation.

**Figure 8:**
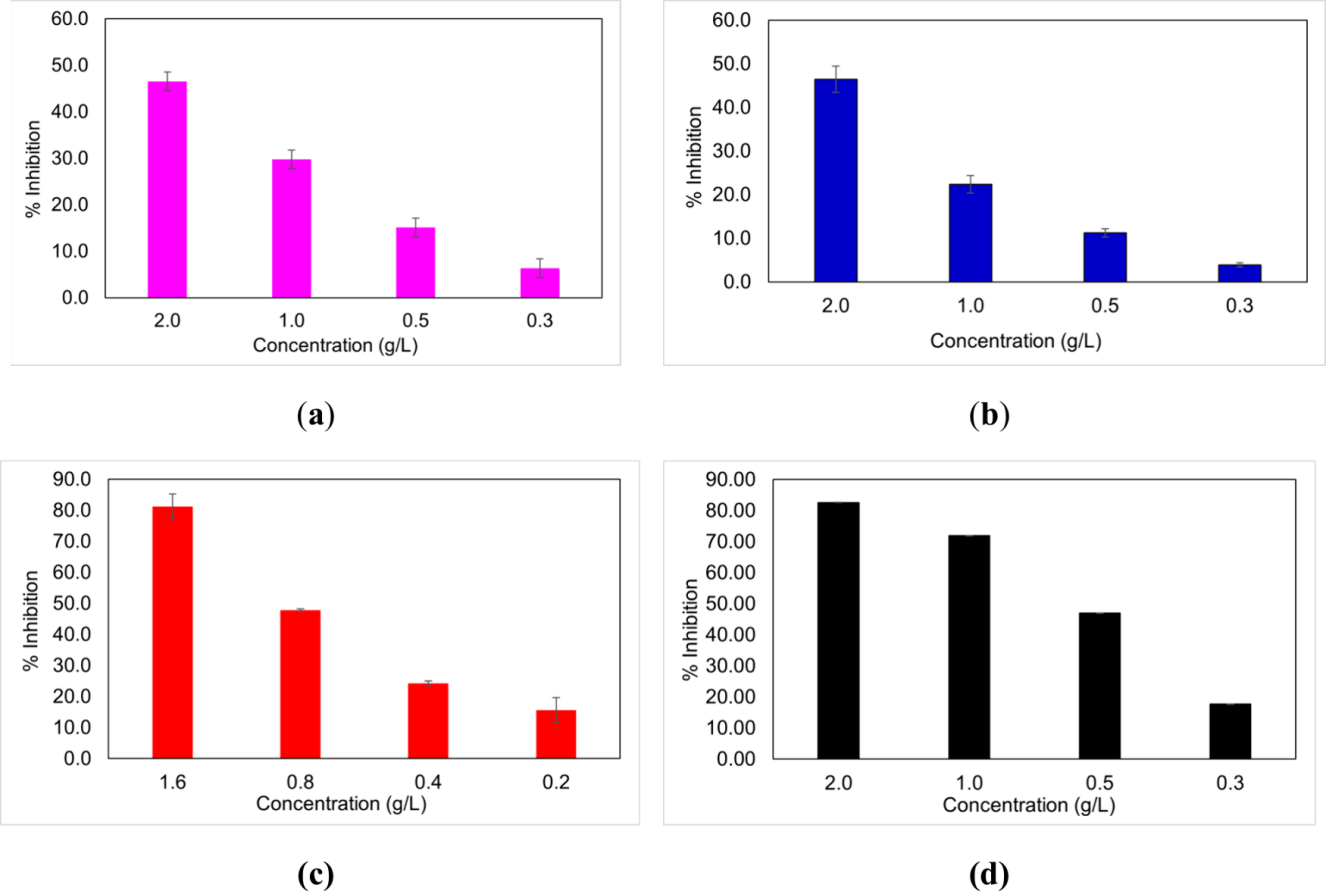
ABTS antioxidant activity assay of fucoidan-coated liposomes (a) before and (b) after irradiation, empty liposomes (c) before and (d) after photodegradation. The IC50 was as follows for fucoidan-coated liposomes (2.0 ± 0.6) g/L and (2.0 ± 0.1) g/L, and empty liposomes (0.9 ± 0.4) g/L and (0.9 ± 0.6) g/L, respectively. Statistical analysis revealed significant differences (*p* < 0.05); however, the impact was less than 5%, ensuring the maintenance of structural integrity.

Before photodegradation, fucoidan-coated liposomes (2 g/L) exhibited a maximum scavenging capacity of 47 ± 3%, while after irradiation, the activity decreased slightly to 46 ± 3%. In contrast, empty liposomes before photodegradation showed that 1.6 mg/mL had the best scavenging effect 81 ± 4%, and after degradation, we observed that 2 mg/mL had a scavenging effect of 82.62 ± 0.03%. The IC_50_ values for fucoidan-coated liposomes were 2.0 ± 0.6 and 2.0 ± 0.1 g/L, before and after photodegradation, respectively, corresponding to an AAE of 39 ± 10 μmol/g. For empty liposomes, the IC_50_ values were (0.9 ± 0.4) and (0.9 ± 0.6) g/L, with corresponding AAE values of 86 ± 40 and 86 ± 60 μmol/g (Table 3).

**Table 3:** Antioxidant Activity of Empty and Fucoidan-Coated Liposomes Expressed as Inhibitory Concentration Values (g/L) and Ascorbic Acid Equivalents (μmol/g) for Each Assay

formulation	time	IC_50_ (g/L)	AAE (μmol/g)
FDL	0 min	2.0 ± 0.6	39 ± 10
FDL	30 min	2.0 ± 0.1	39 ± 10
EMPL	0 min	0.9 ± 0.4	86 ± 40
EMPL	30 min	0.9 ± 0.6	86 ± 60

Antioxidant assays revealed that empty liposomes exhibited slightly higher ABTS radical scavenging activity than fucoidan-coated formulations (Figure 8). This difference (<5%) was statistically significant but not biologically meaningful. At first glance, this result appears counterintuitive given the well-documented antioxidant properties of fucoidan, which arise from its sulfated polysaccharide backbone and capacity to donate electrons or hydrogen atoms.^67^ Several physicochemical considerations may reconcile this observation.

The most plausible mechanism involves steric and diffusional hindrance at the vesicle interface. The fucoidan coating forms a hydrated polyanionic shell around the liposomes, which increases colloidal stability but simultaneously limits the diffusion of ABTS radicals to the lipid core where redox-active sites reside. This results in a lower apparent radical scavenging rate, even though the system itself remains chemically more stable. Similar diffusion-limited effects have been reported for polysaccharide- and polymer-coated nanocarriers, where enhanced stability is accompanied by reduced accessibility of embedded antioxidants.^68^ Additionally, fucoidan-lipid interactions may alter the orientation or availability of reactive functional groups (such as hydroxyls and carbonyls) at the bilayer surface, thereby modulating the kinetics of radical quenching. The phospholipid bilayer in empty liposomes also contains intrinsic antioxidants, such as unsaturated fatty acids and tocopherols, that contribute directly to ABTS•+ reduction, further explaining their slightly higher measure of activity.

This apparent discrepancy is further clarified by the compositional and structural stability revealed in FTIR and GC-MS analysis. FTIR spectra of fucoidan-coated liposomes displayed characteristic adsorption bands, which confirm the abundance of hydroxyl and sulfate functional groups capable of electron donation and radical stabilization. Meanwhile, GC-MS analysis of the lipid matrix demonstrated that fucoidan-coated liposomes retained significantly higher levels of fatty acids after photodegradation compared with empty liposomes. This suggests that fucoidan provides indirect antioxidant protection by preventing lipid peroxidation and oxidative degradation rather than directly scavenging free radicals. Thus, the lower ABTS does not reflect weaker antioxidant capacity but instead restricted radical accessibility due to the fucoidan barrier.

In essence, FTIR confirms the presence of functional groups responsible for electron transfer and radical quenching, while GC-MS reveals the preserved lipid integrity under oxidative stress. Together, these data demonstrate that fucoidan’s antioxidant mechanism is barrier-mediated and structural, effectively limiting oxidative chain propagation within the lipid bilayer despite apparently lower ABTS reactivity.^28,69^

When analyzed collectively, these observations suggest that the antioxidant mechanism of fucoidan-coated liposomes is indirect but sustained and relies on structural stabilization, radical exclusion, and prevention of lipid oxidation rather than immediate chemical scavenging. Although the numerical antioxidant indices appear higher in empty liposomes, they reflect only short-term redox kinetics and do not capture long-term photoprotective performance. The FTIR, GC–MS, and thermal analyses collectively demonstrate that fucoidan confers superior oxidative and structural stability, highlighting its relevance as a functional coating for prolonged antioxidant efficacy in liposomal applications.

Although the differences in ABTS activity between empty and fucoidan-coated liposomes were statistically significant, their magnitude was relatively small (<5%). From a formulation perspective, such minor variations are unlikely to yield measurable differences in biological performance in skin applications. Moreover, chemical antioxidant indices do not necessarily correlate with biological performance, which depends more on colloidal stability, photoprotection, and sustained antioxidant release. In this regard, fucoidan-coated liposomes are expected to outperform uncoated systems because the fucoidan layer provides resistance to aggregation and degradation under oxidative or photolytic stress. Consequently, the slight reduction in ABTS activity should not be interpreted as a loss of functionality but as a methodological artifact of interfacial diffusion. Such stability and prolonged protection make fucoidan-coated liposomes promising candidates for cosmetic and pharmaceutical formulations exposed to oxidative or environmental stress.

### 3.8 Mechanistic Insight: Fucoidan-Lipid Bilayer Interactions

The enhanced stability, functionality, and bioactivity observed in fucoidan-coated liposomes can be attributed to specific physicochemical interactions between the fucoidan polysaccharide and omega-rich algal lipids. Fucoidan is a sulfated, anionic polysaccharide rich in L-fucose and sulfate ester groups, which play a central role in its ability to form stable nanostructures.^70^ Similar structure–function relationships have been observed in other natural extracts containing hydroxyl- and carboxyl-rich biomolecules, where hydrogen bonding and electrostatic interactions contribute to radical scavenging and anti-inflammatory effects.^71–73^ Such correlations suggest that the stabilization mechanisms identified in fucoidan-coated liposomes may be extended to other polysaccharide- or phenolic-rich systems, reinforcing the universality of hydrogen-bond-driven and electrostatically enhanced antioxidant behavior.

One of the principal stabilization mechanisms is electrostatic interaction: the negatively charged sulfated groups of fucoidan interact with the positively charged or zwitterionic head groups of phospholipids. This association increases the surface charge (as reflected in a more negative zeta potential) and promotes electrostatic repulsion that prevents vesicle aggregation and improves the colloidal stability. For instance, fucoidan-coated liposomes exhibit zeta potentials ranging from −35 to −50 mV, indicating superior electrostatic stabilization compared to other polymers.^34^ In addition, hydrogen bonding may occur between the hydroxyl groups of fucoidan and the polar head groups or water molecules surrounding the lipid bilayer. These interactions can stabilize the liposome surface by forming a hydration shell that resists collapse or fusion. Fucoidan may also adsorb onto or intercalate within the outer layer of the bilayer through van der Waals interactions and hydrophobic effects, contributing to membrane integrity and reduced permeability.

Together, these interactions result in increased steric stabilization, reduced oxidative degradation, and enhanced photothermal resistance of the liposomal system, making this system highly suitable for dermatological and skincare applications.

### 3.9 Comparative Analysis: Fucoidan-Coated Liposomes vs Other Natural-Based Carriers

Fucoidan-coated liposomes offer significant advantages for skincare applications over other natural-based carriers, such as chitosan and alginate, due to their stability and functionality (Table 4). This was reflected in our comparative analysis (Figure 9), where fucoidan-coated systems showed the strongest negative surface charge, lowest PDI, and highest photostability. These parameters directly translate to improved colloidal stability and resistance to UV-induced degradation, enabling the controlled release of encapsulated actives under oxidative or light stress conditions. The sustained negative charge across a wide pH range further supports long-term suspension stability, consistent with previous storage studies (ζ ≈ −72 mV over 28 days). Additionally, fucoidan itself possesses bioactive properties, including anti-inflammatory, antioxidant, and skin-regenerative effects, which make it beneficial as a stabilizing agent and as a functional ingredient.^25^

**Table 4:**
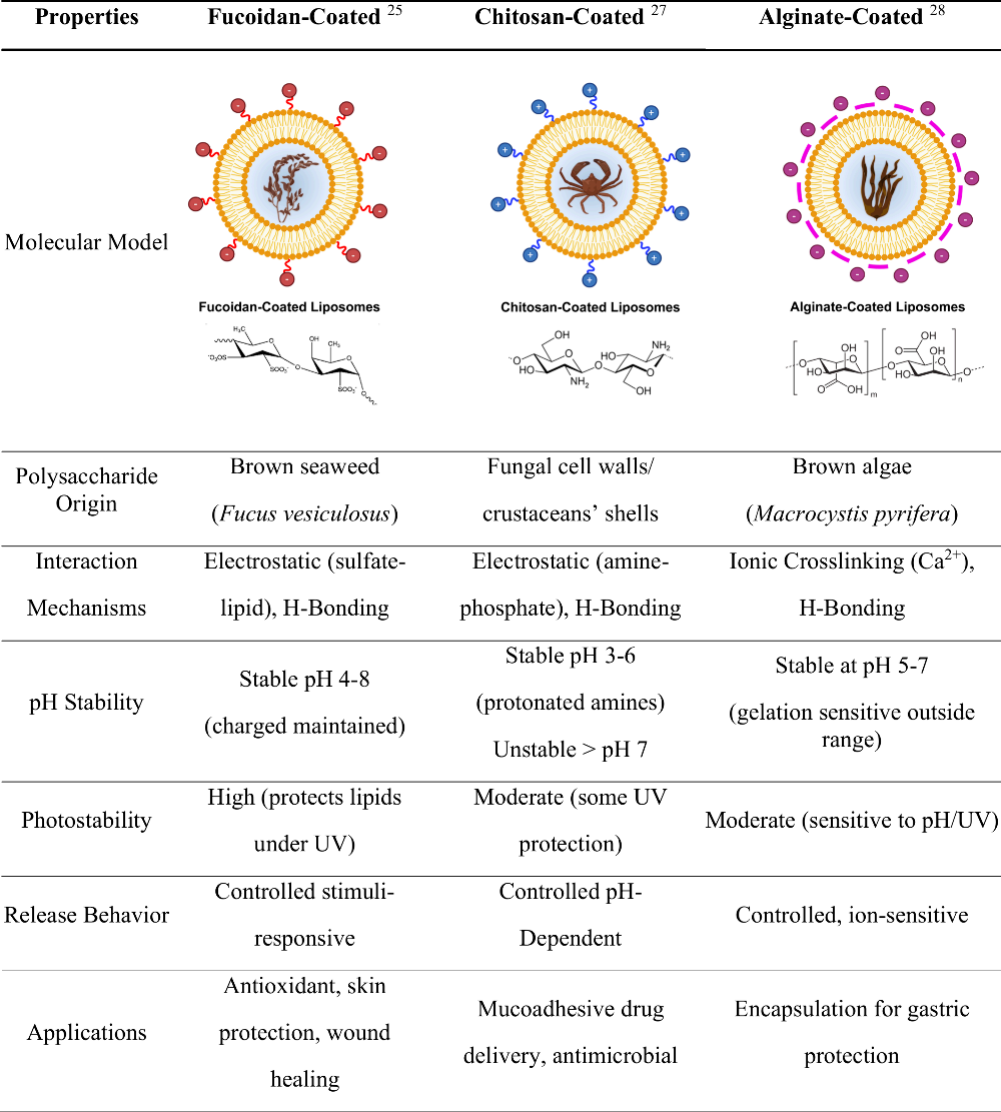
Evaluation of Natural Polysaccharide-Based Liposomes: Origin, Mechanism, Stability, and Drug Delivery Performance^a^

a Image created in BioRender.

**Figure 9:**
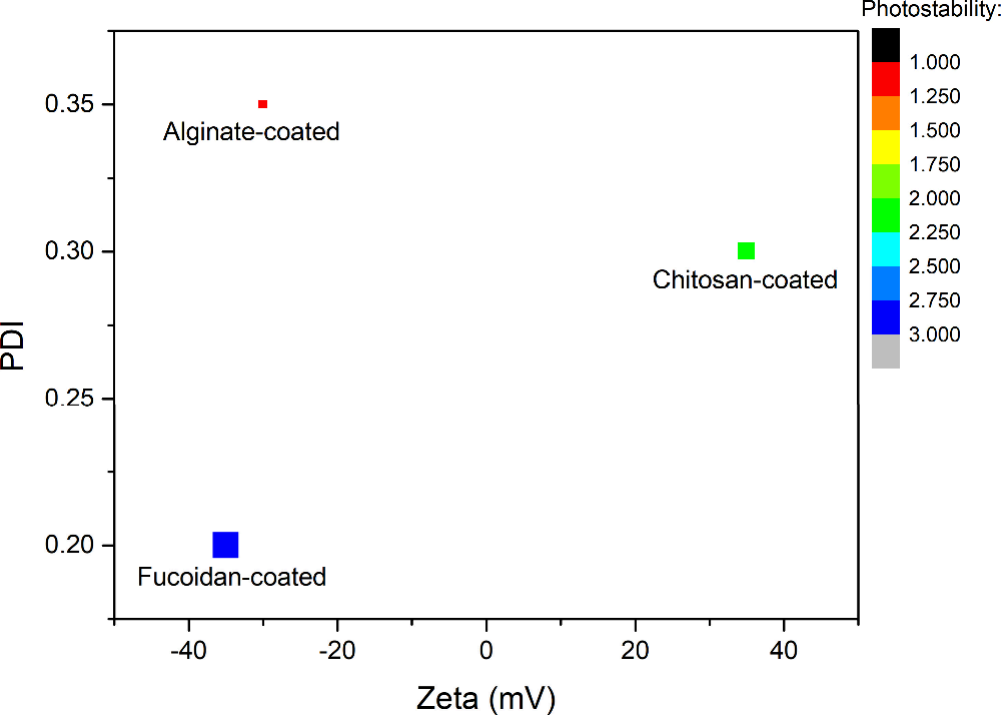
Materials-selection bubble map showing the relationship between the zeta potential and PDI for fucoidan-, alginate-, and chitosan-coated nanoparticles. Bubble color and size represent relative photostability values. Fucoidan-coated particles exhibited the lowest PDI and best photostability (the bigger the square, the better the stability).

The literature highlights chitosan and alginate as two of the most extensively studied natural polysaccharides for liposome stabilization, primarily due to their biocompatibility, mucoadhesiveness, and contrasting charge profiles that can enhance liposomal stability and prolonged shelf life. Chitosan, a cationic polysaccharide, improves the stability, integrity, and mucoadhesive properties of liposomes, promoting cellular uptake and resistance to aggregation and leakage under physiological conditions. However, its positive charge can also induce charge reversal at neutral pH, leading to aggregation and burst release due to decreased homogeneity, as reflected by a higher PDI and intermediate photostability.^27^ In contrast, alginate, an anionic polysaccharide characterized by excellent gel-forming capacity and biocompatibility, stabilizes liposomes primarily through ionic cross-linking and supports controlled drug release. However, alginate coatings may generate thicker interfacial layers that impede diffusion and decrease encapsulation efficiency, reflected in elevated PDI values and diminished photostability.^28^

Fucoidan, with its lower molecular weight and flexible conformation, forms a thinner yet more functional coating that preserves the lipid integrity and allows for controlled release, especially under oxidative or UV-induced stress. Moreover, fucoidan possesses inherent bioactive properties, including antioxidant, anti-inflammatory, and skin-regenerating effects that are not commonly attributed to chitosan or alginate. This dual role (as a structural stabilizer and active agent) makes fucoidan-coated liposomes particularly suitable for skincare and dermatological formulations, where prolonged photoprotection and biocompatibility are essential.

## 4 Conclusions

This study demonstrated that fucoidan-coated liposomes derived from *Sargassum* exhibited improved physicochemical stability and resilience under photo- and thermal-stress conditions, positioning them as promising candidates for sustainable skincare formulations. While the work establishes a strong foundation through structural and stability characterization, further research is needed to validate biological efficacy, including cytotoxicity, skin penetration, and photoprotective assays in cell models. Future studies should also investigate long-term stability, release kinetics, formulation compatibility, and scale-up potential to advance the translation of these compounds into practical, multifunctional skincare applications. In comparison with prior studies, this strategy uniquely combines marine biomass valorization with improved liposomal stability and antioxidant capacity, establishing fucoidan-coated *Sargassum* liposomes as a durable and eco-conscious platform for future skincare formulations within a circular economy paradigm. By addressing these research gaps, fucoidan-coated liposomes derived from *Sargassum* can be more comprehensively positioned as a sustainable and effective platform for next-generation skincare technologies.

## Supplementary Material



## Notes

GC–MS chromatograms of empty and fucoidan-coated liposomes before and after photodegradation (PDF)

## Abbreviations

UV: ultraviolet radiation
Tm: main transition temperature
DLS: dynamic light scattering
PDI: polydispersity index
FTIR: Fourier-transform infrared spectroscopy
ATR: attenuated total reflectance
DSC: differential scanning calorimetry
TGA: thermal gravimetric analysis
ABTS: 2,2′-azino-bis­(3-ethylbenzothiazoline-6-sulfonic acid)
ANOVA: analysis of variance
FDL: fucoidan-coated liposomes
EMPL: empty liposomes
FD: fucoidan
PA: palmitic acid
AA: arachidonic acid
OA: oleic acid
