# Supplementary material for: Photoprotective and Antioxidant Liposomal Formulations from Sargassum-Derived Biomolecules for Skincare Applications
Source: ACS Omega. 2025 Oct 25;10(43):52018–31. doi: 10.1021/acsomega.5c09406 (PMC12593078; doi:10.1021/acsomega.5c09406)
Supplement: Supplementary file 1 [file ao5c09406_si_001.pdf]

Supporting Information:

Photoprotective and Antioxidant Liposomal Formulations from Sargassum-Derived Biomolecules for Skincare Applications

Angela Cruz-Lugo, Sofía V. Rivera-Santiago, Ana S. Cortés-Norat, Valeria I. Rosario-Isona, Sandra V. Nieves-Moron, Victoria V. Viera-Sánchez, and Liz M. Díaz-Vázquez**

Author Affiliation:

University of Puerto Rico, Río Piedras Campus, San Juan, 00925-2537, Puerto Rico

Contact Information for Corresponding Authors:

Angela C. Cruz Lugo

Department of Chemistry
University of Puerto Rico-Rio Piedras, San Juan 00925-2537, Puerto Rico
Email: angela.cruz@upr.edu

Liz M. Díaz Vázquez

Department of Chemistry
University of Puerto Rico-Rio Piedras, San Juan 00925-2537, Puerto Rico
Email: liz.diaz2@upr.edu

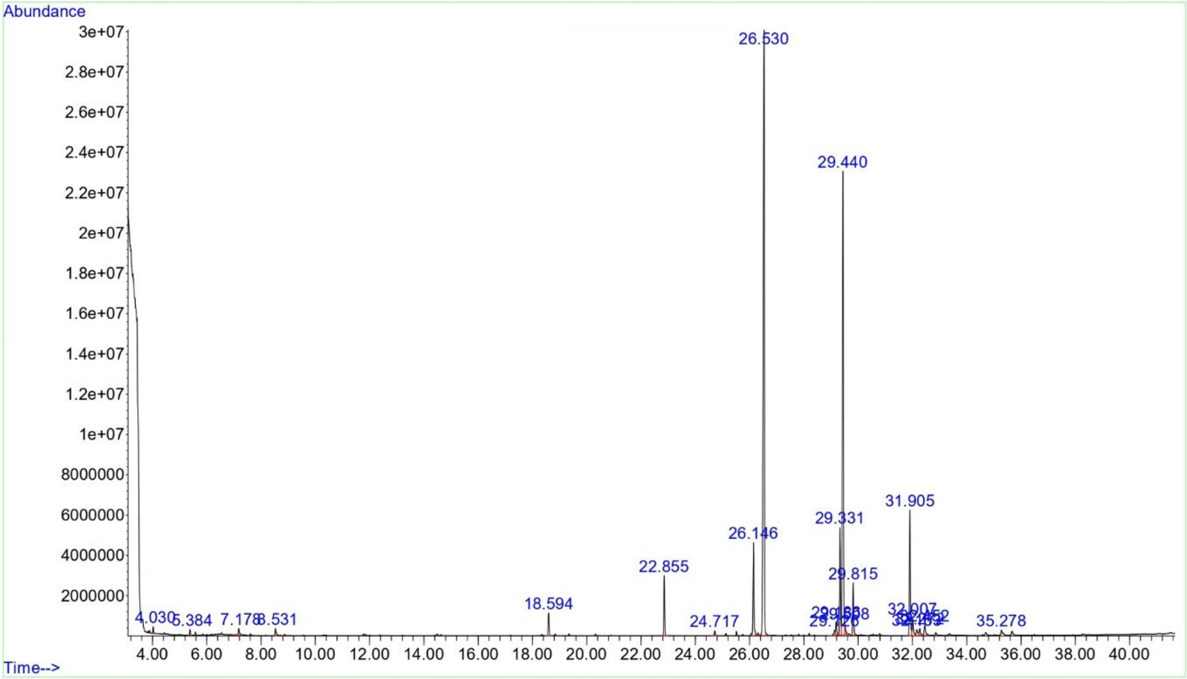

FIGURE S1: GC chromatogram of fucoidan-coated liposomes pre-photodegradation (0 mins), showing retention times of detected components.

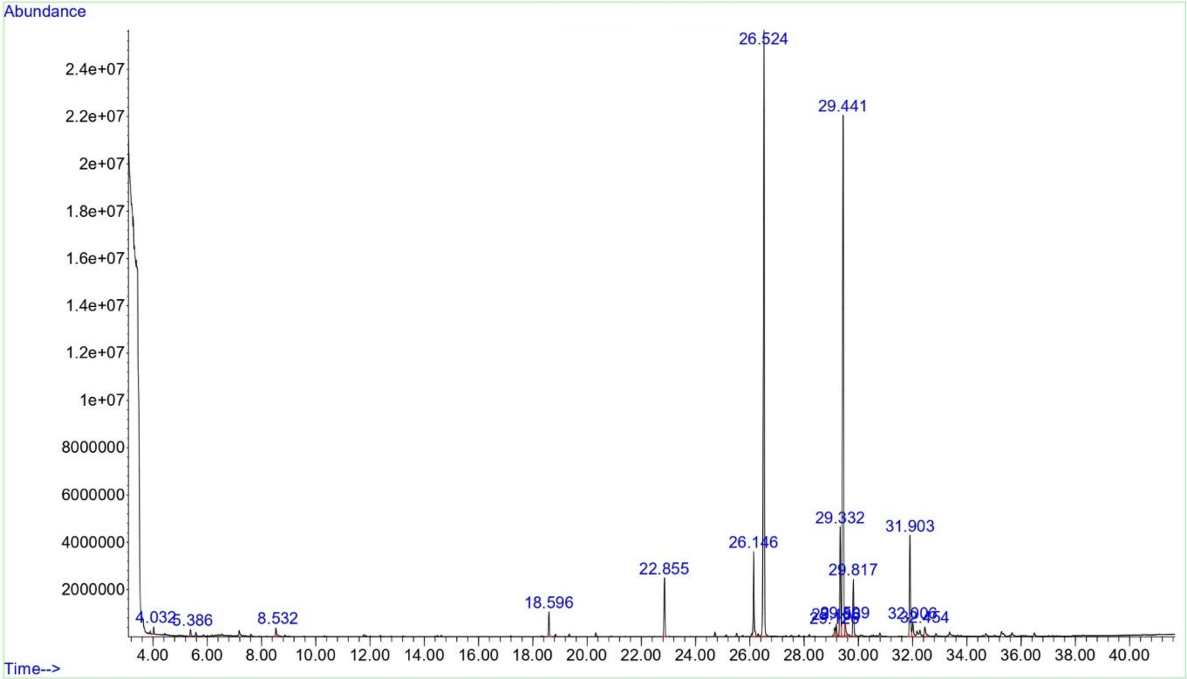

FIGURE S2: GC chromatogram of fucoidan-coated liposomes post-photodegradation (30 mins), showing retention times of detected components.

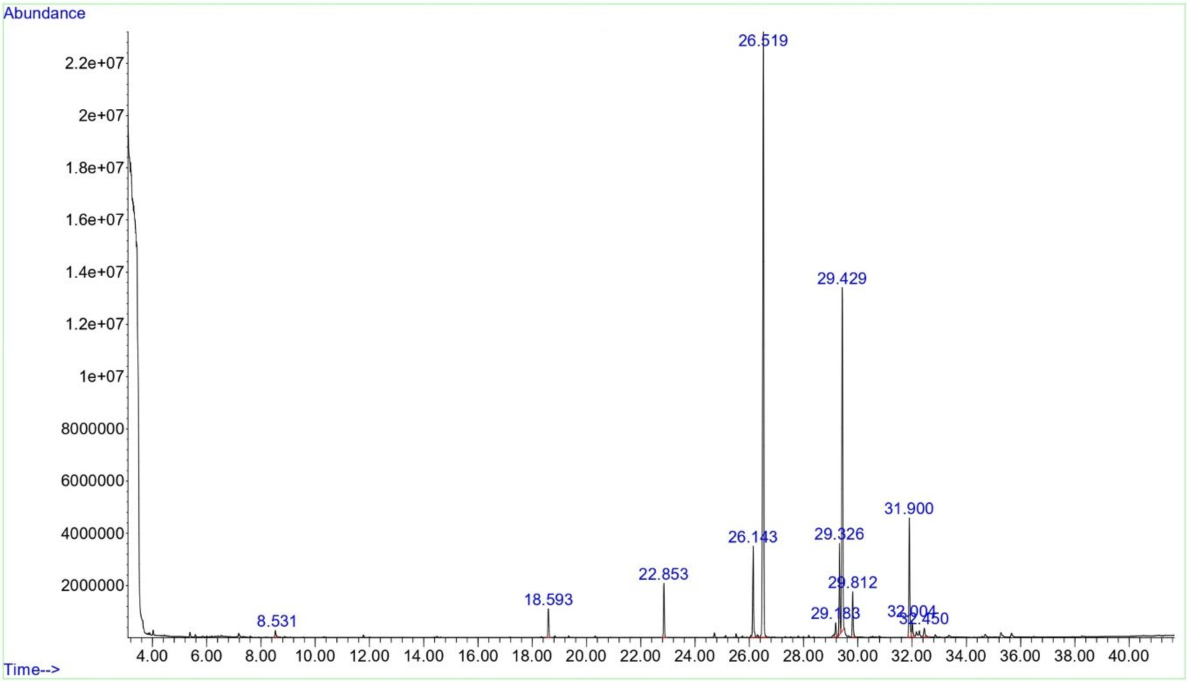

FIGURE S3: GC chromatogram of empty liposomes pre-photodegradation (0 mins), showing retention times of detected components.

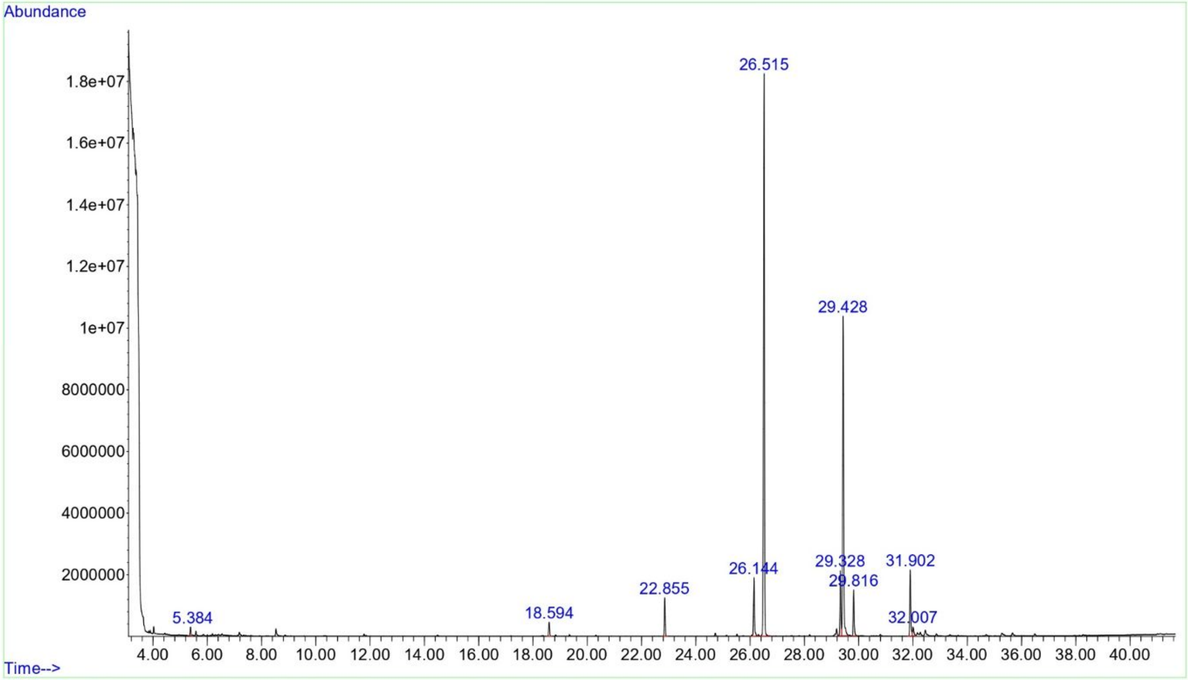

FIGURE S4: GC chromatogram of empty liposomes post-photodegradation (30 mins), showing retention times of detected components.
